# β-Glucan Derived from *Aureobasidium pullulans* Is Effective for the Prevention of Influenza in Mice

**DOI:** 10.1371/journal.pone.0041399

**Published:** 2012-07-23

**Authors:** Daisuke Muramatsu, Atsushi Iwai, Shiho Aoki, Hirohumi Uchiyama, Koji Kawata, Yosuke Nakayama, Yasuhiro Nikawa, Kisato Kusano, Mitsuyasu Okabe, Tadaaki Miyazaki

**Affiliations:** 1 Aureo Science Co., Ltd., Sapporo, Hokkaido, Japan; 2 Aureo Co., Ltd., Kimitsu, Chiba, Japan; 3 Department of Bioresources, Hokkaido University Research Center for Zoonosis Control, Sapporo, Japan; 4 Division of Molecular Immunology, Institute for Genetic Medicine, Hokkaido University, Sapporo, Japan; 5 Research Section of Probiotics Immunology, Institute for Genetic Medicine, Hokkaido University, Sapporo, Japan; Lovelace Respiratory Research Institute, United States of America

## Abstract

β-(1→3)-D-glucans with β-(1→6)-glycosidic linked branches produced by mushrooms, yeast and fungi are known to be an immune activation agent, and are used in anti-cancer drugs or health-promoting foods. In this report, we demonstrate that oral administration of *Aureobasidium pullulans*-cultured fluid (AP-CF) enriched with the β-(1→3),(1→6)-D-glucan exhibits efficacy to protect mice infected with a lethal titer of the A/Puerto Rico/8/34 (PR8; H1N1) strain of influenza virus. The survival rate of the mice significantly increased by AP-CF administration after sublethal infection of PR8 virus. The virus titer in the mouse lung homogenates was significantly decreased by AP-CF administration. No significant difference in the mRNA expression of inflammatory cytokines, and in the population of lymphocytes was observed in the lungs of mice administered with AP-CF. Interestingly, expression level for the mRNA of virus sensors, RIG-I (retinoic acid-inducible gene-I) and MDA5 (melanoma differentiation-associated protein 5) strongly increased at 5 hours after the stimulation of *A. pullulans*-produced purified β-(1→3),(1→6)-D-glucan (AP-BG) in murine macrophage-derived RAW264.7 cells. Furthermore, the replication of PR8 virus was significantly repressed by pre-treatment of AP-BG. These findings suggest the increased expression of virus sensors is effective for the prevention of influenza by the inhibition of viral replication with the administration of AP-CF.

## Introduction

β-glucans are polysaccharides which consist of glucose chains linked with β-glycosidic bonds. Especially, β-glucans which consist of a β-(1→3)-D-glycosidic linked main chain and β-(1→6)-D-glycosidic linked branches have immune modulation effects, and the beneficial effects of the β-glucans for promoting health when taken as a supplement are believed [1]–[3]. Previous studies demonstrated that β-(1→3),(1→6)-D-glucans exhibit anti-tumor [4]–[6], anti-infectious disease [7] and anti-inflammatory [8], [9] activities through the modulation of the immune system. Further, β-(1→3),(1→6)-D-glucan-containing agents which are produced by mushrooms and fungi, such as krestin [10], picibanil [11], lentinan [12], and sizofiran [13], are actually used as anti-cancer drugs.

A black yeast, *Aureobasidium pullulans*, extracellularly produces a β-(1→3),(1→6)-D-glucan highly branched with β-(1→6)-glycosidic bonds at a certain condition with high efficiency [14]–[16]. The β-(1→3),(1→6)-D-glucan contained in the cultured fluid of *A. pullulans* is approved as a food additive, and is consumed as a health-promoting food in many countries. Basically, an extraction process, such as hot-water extraction, is required for the production of the β-(1→3),(1→6)-D-glucan-containing supplemental food derived from other organisms. On the other hand, the β-(1→3),(1→6)-D-glucan produced by *A. pullulans* is yielded in a water-soluble form as a viscous liquid, and is ready to use as a food additive without extraction. In addition to this advantage, the *A. pllulans*-produced β-(1→3),(1→6)-D-glucan has almost the same efficacy as this type of glucan produced by other organisms. For instance, the anti-tumor [17], [18], anti-infectious disease [19] and anti-allergic [20] activities of the β-(1→3),(1→6)-D-glucan produced by *A. pllulans* have been reported.

Influenza is known to be an acute respiratory tract disease caused by the infection of an influenza virus [21], [22]. Influenza epidemics occur almost every winter, and the social and economic damage caused by influenza is a considerable problem in many countries. Although influenza is frequently accompanied by hyperthermia with arthralgia and/or ague, usually, in healthy adults, it would rarely induce a lethal case, but in many cases patients spontaneously recover from influenza without antiviral drug treatment. However, influenza virus infection is quite dangerous to a certain percentage of the population, such as pregnant women, diabetes patients, infants and elderly people, who are known to be a high risk group [23], [24]. Since these people have some deficiency for immune responses, the viral infection of this high risk group frequently leads to severe and sometimes lethal cases. Therefore, maintaining the immune system in an appropriate condition is thought to be important for the prevention of severe symptoms of influenza.

In this study, we focus on the immune stimulatory function of the *A. pullulans*-produced β-(1→3),(1→6)-D-glucan, and demonstrate that oral administration of *A. pullulans*-cultured fluid (AP-CF) enriched with the β-(1→3),(1→6)-D-glucan exhibits efficacy to increase the survival rate of mice infected with the A/Puerto Rico/8/34 (PR8; H1N1) strain of influenza virus. The oral administration of AP-CF protects the mice from a lethal PR8 virus challenge, and the virus titer in the lung homogenates significantly decreased by AP-CF administration 3 days after the virus infection. The stimulation of purified *A. pullulans*-produced β-(1→3),(1→6)-D-glucan (AP-BG) induces the expression of virus sensors, RIG-I (retinoic acid-inducible gene-I) and MDA5 (melanoma differentiation-associated protein 5) mRNAs 5 hours after stimulation in a macrophage-derived cell line, RAW264.7 cells [25] and macrophages, differentiated THP-1 cells [26], [27]. In addition, the replications of the PR8 virus in RAW2674 cells are significantly inhibited by treatment with AP-BG. These results indicate a possible mechanism whereby the oral administration of AP-CF exhibits efficacy for the prevention of influenza by the inhibition of virus replication.

## Results

### Oral administration of A. pullulans-cultured fluid (AP-CF) increases the survival rate of mice after a lethal influenza A virus infection

**Figure 1:**
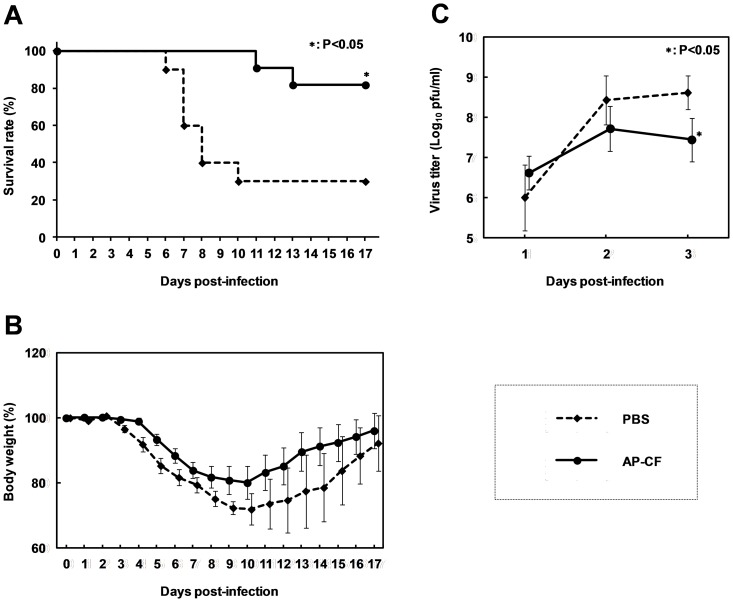
The oral administration of Aureobasidium pullulans-cultured fluid (AP-CF) protects mice from lethal PR8 virus infections. (A, B) AP-CF (40 µg/mouse, n = 11), or PBS (n = 10) was orally administered to the C57BL/6N mice throughout the experiment once a day, from 7 days before the virus infection. After the 7 day pre-treatment, the mice were intranasally infected with the A/PuertoRico/8/34 (PR8; H1N1) strain of influenza A virus at a titer of 2,000 pfu, and then the survival rate (A) and the body weight loss (B) of the mice were recorded. Error bars indicate the standard error of the mean. (C) The mice (n = 5 for each group) were infected with influenza A virus at the same condition as in panel A and B, and then the lungs of the mice were collected at the day indicated in the figure. Virus titers in the lung homogenates were measured by plaque assay. The error bars indicate standard deviations which were calculated by five independent experiments. An asterisk (*) indicates P<0.05 compared with the mice treated with PBS.

To investigate the effect of oral administration of *Aureobasidium pullulans*-cultured fluid (AP-CF) on the prevention of influenza, we used the A/Puerto Rico/8/34 (PR8; H1N1) strain, a laboratory strain which exhibits high virulence for C57BL/6N mice. The mice were orally administered with AP-CF or PBS once a day for 7 days, and subsequently were infected with the PR8 strain of influenza virus in a titer of 2,000 pfu. Oral administration of AP-CF or PBS was continued once a day until day 17, and the body weight losses and clinical observations of the mice were monitored. The results show that the survival rate of the mice orally administered with AP-CF significantly increased in comparison with that of the control mice (Figure 1A).

To investigate the effect of oral administration of AP-CF on the replication of the PR8 strain of influenza virus, we monitored virus titers in the lungs by plaque assay. As shown in Figure 1C, the results indicate that although the virus titers in the lung were not different between AP-CF administered mice and control mice on day 1, the virus titers significantly decreased in AP-CF administered mice on the day 5 after the virus infection.

### Oral administration of AP-CF did not significantly affect to the expression of inflammatory cytokines in the lungs of mice infected with the influenza A virus

**Figure 2:**
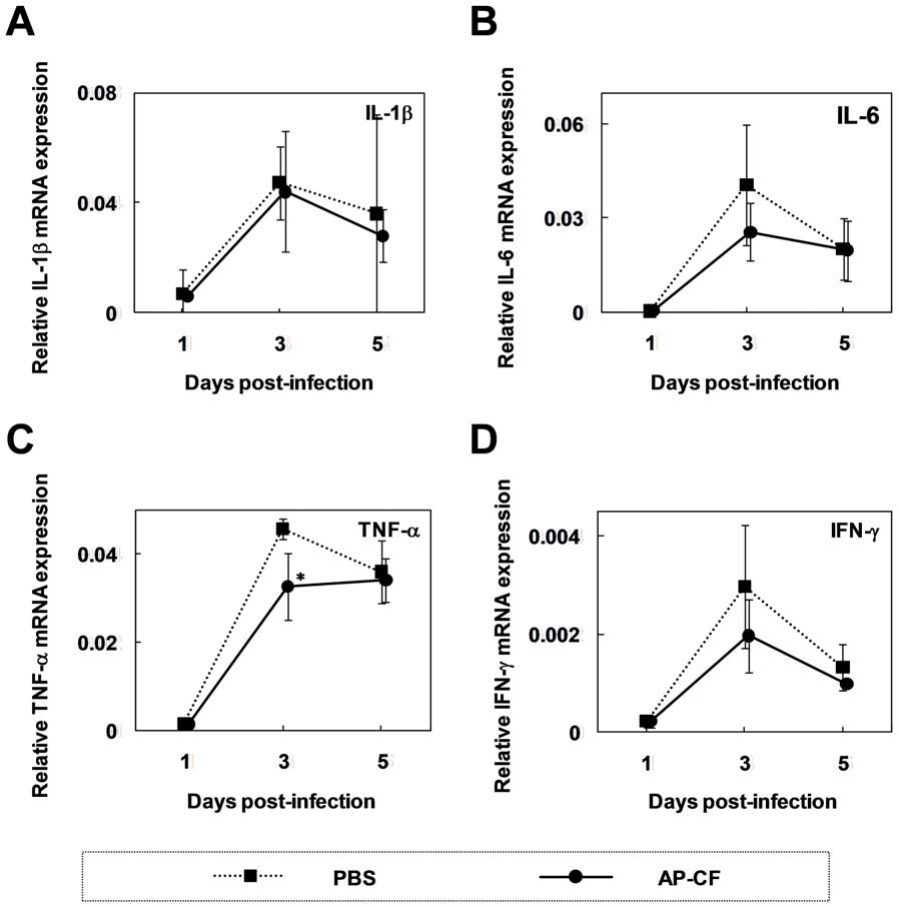
The oral administration of AP-CF inhibits influenza A virus replication in the lung. Total RNAs were isolated from the lung tissue of mice infected with influenza A virus at the day indicated in each panel. Subsequently, the RNAs were subjected to real-time RT-PCR analysis using a specific primer set for interleukin-1β (IL-1β; A), IL-6 (B), tumor necrosis factor-α (TNF-α; C), or interferon-γ (IFN-γ; D). The data represent relative mRNA expressions which were normalized with the expression level of glyceraldehyde-3-phosphate dehydrogenase (G3PDH) mRNA. Error bars indicate standard deviations which were calculated with three independent experiments. An asterisk (*) indicates P<0.05 compared with the control mice.

To understand the mechanism for the protection of the mice from a sublethal titer infection of the influenza A virus by oral administration of AP-CF, we performed real-time RT-PCR to analyse the change of gene expression in the mouse lung. Abnormal production of inflammatory cytokines is known to be frequently found in the course of lethal influenza virus infection, and is thought to be linked with disease severity [28]–[31]. Thus, we investigated the effects of orally administered AP-CF on the expression of several inflammatory cytokines. As shown in Figure 2, the results show that there is a tendency that the mean expression levels of all inflammatory cytokines, interleukin-1β (IL-1β), IL-6, tumor necrosis factor-α (TNF-α), and interferon-γ (IFN-γ), slightly decreased with oral administration of AP-CF. However, the differences were not statistically significant except TNF-α mRNA at the day 3, and the decrement of TNF-α mRNA expression was only fewer than 1.5 folds. Further, to investigate the effect of oral administration of AP-CF on Type I interferon (IFN) expression, the mice were orally administered with AP-CF for 7 days, and the expression of interferon IFN-α in the serum was monitored by ELISA. The data indicated that the expression of IFN-α slightly tended to increase in the serum from the mice orally administered with AP-CF (data not shown). However, the increment level of serum IFN-α was not statistically significant. Therefore, the effects of the orally administered AP-CF on the production of inflammatory cytokines are thought to be quite weak.

### The Population of lung immune cells except DC cells in mice infected with the influenza A virus is not significantly affected by the oral administration of AP-CF

**Figure 3:**
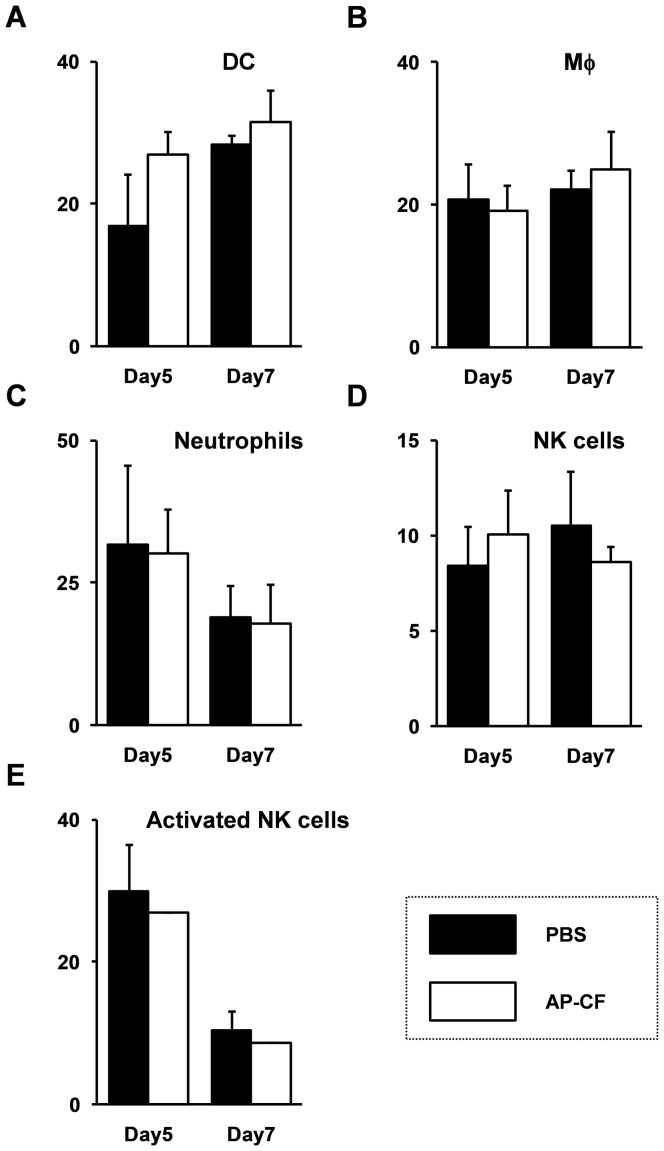
The population of lung immune cells except DC cells is not significantly affected by the oral administration of AP-CF after an influenza A virus infection. The C57BL/6N mice were infected with the influenza A virus at the same condition as in Figure 1, and then the whole lung tissues were extracted from the mice. The cells were dissociated by the treatment with collagenase and DNaseI, then stained with fluorescent labeled specific antibodies and analyzed by the FACS (FACS Canto; BD bioscience). The data represent the percentage of the cells from total alive cells (7-AAD negative cells). Error bars indicate standard deviations which were calculated with three independent experiments. (A) dendritic cell (DC); CD11c^+^, F4/80^−^; (B) macrophage (Mφ): F4/80^+^; (C) neutrophills: Gr-1^+^; (D) natural killer (NK) cell: CD49b^+^, CD3^−^; (E) activated NK cell: CD49b^+^, CD3^−^, CD69^+^.

Since no apparent effect of orally administered AP-CF on the mRNA expressions of inflammatory cytokines has been found, we investigated the effects of AP-CF on the population of lung lymphocytes with flow cytometric analysis. The mice orally administered with AP-CF were infected with the PR8 strain of influenza virus, and on day 5 or 7 post-infection, the mice lungs were extracted. The populations of the lung lymphocytes were fluoro-stained with some antibodies and analyzed by FACS (FACS Canto, BD bioscience). The results show that in addition to the effect on the induction of pro-inflammatory cytokines, the population of lymphocytes in the lungs of AP-CF administered mice was not significantly changed in comparison with that of the control mice (Figure 3A–D). The population of dendritic cells in the lungs tended to slightly increase with administration of AP-CF on day 5 after influenza A virus infection (Figure 3A). However, the difference was not statistically significant. Further, the activation of natural killer cells was not induced by the oral administration of AP-CF (Figure 3E). These results demonstrate that oral administration of AP-CF exhibits efficacy to inhibit the replication of influenza A virus without strong systemic effects on the population of these immune cells.

### Production of growth factors and inflammatory cytokines are induced by stimulation with purified β-D-glucan in macrophages

**Figure 4:**
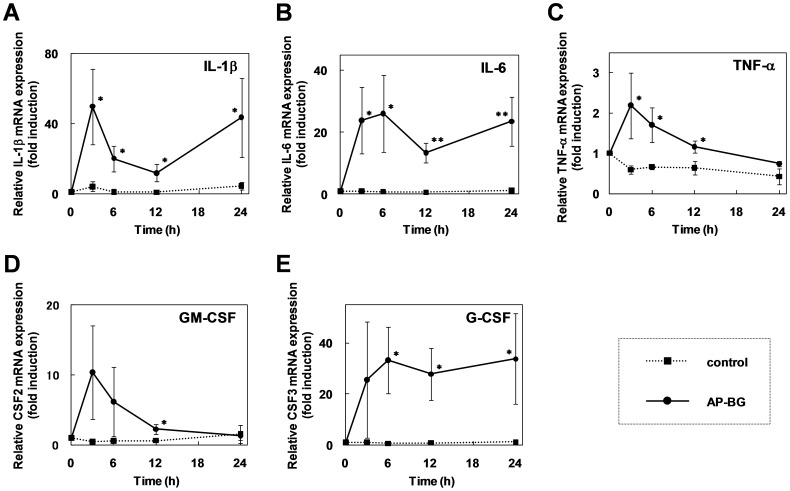
Stimulation with purified Aureobasidium pullans-produced β-(1→3),(1→6)-D-glucan (AP-BG) induces the production of growth factors and inflammatory cytokines in RAW264.7 cells. (A–E) RAW264.7 cells were stimulated with AP-BG in a concentration of 100 µg/ml, and then the cells were harvested at some time points of post-stimulation indicated in the figure. Total RNAs isolated from the cells were then analyzed by real-time RT-PCR using specific primer sets for interleukin-1β (IL-1ß; A), IL-6 (B), tumor necrosis factor-α (TNF-α; C), granulocyte-macrophage colony-stimulating factor (GM-CSF; D), and granulocyte colony-stimulating factor (G-CSF; E). The expression of genes was normalized with the expression of G3PDH mRNA, and the data represent a relative expression amount compared with the initial time point. Error bars indicate standard deviations which were calculated with three independent experiments. Single asterisk (*) and double asterisk (**) indicate significant differences between AP-BG-treated cells and control cells with P<0.05 and P<0.01, respectively.

As shown in Figure 2 and Figure 3, the systemic effects of orally administered AP-CF on immunity except the slight increase of DC cell population were hardly demonstrated. Previous reports demonstrated that oral administration of a β-(1→3),(1→6)-D-glucan produced by *A.pullulans* exhibits immunostimulating effects to mice under immune suppressive conditions [32], [33]. However, systemic effects of oral administered the β-(1→3),(1→6)-D-glucan produced by *A.pullulans* on immunity are thought to be mild in healthy individuals, and would be quite difficult to detect. According to our results using mouse model, we thought a possibility that oral administered AP-CF modulates the function of restricted cell lineages, which is undetectable by the analysis of total lung tissue. It has been reported that the stimulation with the β-(1→3),(1→6)-D-glucan activates macrophages [34], [35]. On the other hand, alveolar macrophages (AMs) in lung are known to be important for the initial activation of innate immunity, and crucial for the host defense against influenza A virus infection [36]–[38]. Thus, we focused on the function of macrophage against the influenza A virus infection, and investigated the effect of treatment with the β-(1→3),(1→6)-D-glucan produced by *A.pullulans* on the cultured macrophage derived cells. For the stimulation of the cultured cells, we prepared the purified *A. pullulans*-produced β-(1→3),(1→6)-D-glucan (AP-BG). Our preliminary data showed that the protective activity of orally administered AP-CF against the influenza A virus infection was decreased in mice when the β-(1→3),(1→6)-D-glucan in AP-CF was enzymatically digested with β-glucanase (data not shown). Therefore, the protective effects of orally administered AP-CF against influenza A virus would be mainly dependent on the β-(1→3),(1→6)-D-glucan in AP-CF. Macrophage derived RAW264.7 cells [25] were stimulated with AP-BG, and the time-course for expression of the inflammatory cytokines was analyzed by real-time RT-PCR. As shown in figure 4A–C, the expression of both IL-1β and IL-6 mRNAs was strongly upregulated by the stimulation of AP-BG, whereas expression of TNF-α mRNA was weakly upregulated. In addition, the significant induction was not observed in IFN-γ mRNA after the stimulation with AP-PG (data not shown).

Next, we investigated expression of GM-CSF (granulocyte-macrophage colony-stimulating factor; also called CSF2) and G-CSF (granulocyte colony-stimulating factor; also called CSF3) after the stimulation with AP-GB. These cytokines are known to be essential for proliferation, development and differentiation of lymphocytes from stem cells [39]–[42]. The results show that although the population of the lymphocytes except DC cells in the lungs was not significantly changed by the oral administration of AP-CF (Figure 3), expression of GM-CSF and G-CSF increased in RAW264.7 cells after stimulation with AP-BG (Figure 4D, 4E).

### Expression of RIG-I and MDA5 are transiently induced in macrophages after stimulation of AP-BG

**Figure 5:**
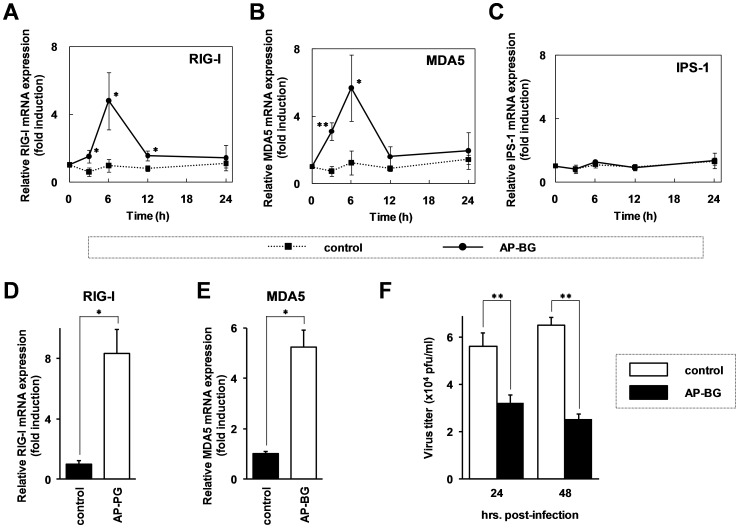
Expression of RIG-I and MDA5 is upregulated in the RAW264.7 cells, after AP-BG stimulation. (A–C) RAW264.7 cells were stimulated with AP-BG at the concentration of 100 µg/ml. At the time point indicated in the figure, the cells were harvested, and the total RNAs isolated from the cells were subjected to real-time RT-PCR analysis using specific primer sets for retinoic acid-inducible gene-I (RIG-I; A), melanoma differentiation-associated protein 5 (MDA5; B), and interferon-β promoter stimulator 1 (IPS-1; C). The data represent relative mRNA expression compared with the expression level of the initial time point, and the calculated values for each time point were normalized with the expression level of G3PDH mRNA. Error bars indicate standard deviations which were calculated by three independent experiments. (D, E) THP-1 cells which were differentiated into macrophages using 100 nM of phorbol 12-myristate 13-acetate (PMA), were stimulated with 100 µg/ml of AP-BG. After 6 hrs, the cells were harvested, and the total RNAs isolated from the cells were subjected to the real-time RT-PCR analysis using specific primer sets for RIG-I (D) and MDA5 (E). (F) RAW264.7 cells were stimulated with AP-BG (100 µg/ml) or PBS for 6 hrs, and then the PR8 strain of influenza A virus was infected to the cells (MOI = 10). After the incubation for the periods indicated in the figure, the virus titers in the cultured medium were measured by plaque assay. Single asterisk (*) and double asterisk (**) indicate significant differences between AP-BG-treated cells and control cells with P<0.05 and P<0.01, respectively.

Type I IFNs, produced by various types of cells, are crucial for activating host cellular anti-virus responses. RIG-I (retinoic acid-inducible gene-I) and MDA5 (melanoma differentiation-associated protein 5) are known to be an intracellular pattern recognition molecule for virus-derived RNAs [43], [44], and are crucial for type I IFNs production in virus infected cells through binding to the mitochondrial adaptor molecule, IPS-1 (interferon-β promoter stimulator 1; also called MAVS, Cardif, VISA). Thus, we investigated the expression of RIG-I, MDA5 and IPS-1 after AP-BG stimulation with real-time RT-PCR. The results show that expression of RIG-I and MDA5 mRNAs increased with AP-BG stimulation, whereas IPS-1 mRNA was not (Figure 5A–C). The previous report demonstrated that RIG-I expression sensitizes to type I IFN response against the influenza A virus infection [45], [46]. Therefore, the induction of RIG-I mRNA after AP-BG stimulation is thought to be an important phenomenon to strengthen the protective activity from influenza A virus infections. Furthermore, the increment of RIG-I and MDA5 mRNA expression was also observed after the AP-BG treatment in the human monocyte derived cell line, THP-1. THP-1 cells were differentiated using phorbol 12-myristate 13-acetate (PMA) into macrophages, and the PMA treated THP-1 cells were then stimulated with AP-BG for 6 hrs. As shown in Figure 5D and 5E, mRNA expression of RIG-I and MDA5 also increased after the stimulation of AP-BG in the PMA-treated THP-1 cells. These results suggest that the AP-BG stimulation upregulates the expression of RIG-I and MDA5 in macrophages.

Next, we investigated replication of the PR8 virus in RAW267.4 cells which were pre-treated with AP-BG. The results demonstrate that the virus titers of influenza A virus in the cultured medium significantly decreased with the pre-treatment of AP-BG (Figure 5F). These observations suggest that increment of RIG-I expression with AP-BG stimulation may inhibit the influenza A virus replication through enhancement of the sensitivity to activate downstream signaling pathways of RIG-I.

## Discussion

In the present study, we demonstrated the protective effects of oral administration of β-(1→3),(1→6)-D-glucan-enriched *Aureobasidium pullulans*-cultured fluid (AP-CF) against the influenza A virus infection. The effect of the oral administration of AP-CF to rescue the mice from the lethal virus infections was apparently indicated by the improvement of the survival rates (Figure 1A) and by decrement of the virus titer in the lungs (Figure 1C). The dose for oral administration of the β-(1→3),(1→6)-D-glucan to the mice in this study is equivalent to the 1 g/day to the humans in the estimation by the body weight. The concentration of β-(1,3),(1,6)-D-glucan in commercially available *Aureobasidium pullulans* cultured fluid is up to 90 mg per sachet. The experimental dose for the mice in this study is equivalent to 11 sachets per day for humans, while the typical dose for daily supplemental food is from 3 to 6 sachets (3 times) per day before meal. Although the experimental dose for the mice in this study is a little higher than the typical dose for humans, the experimental dose is thought to be within the practical applicable range for humans.

In contrast to the protective effects against the influenza A virus infection, the affect of orally administered AP-CF on the expression of pro-inflammatory cytokines, and on the populations of immune cells have not been detected in the lungs infected with the influenza A virus under our experimental conditions (Figure 2 and Figure 3). The *A. pullulans*-cultured fluids are approved as a food additive and have been consumed as a health-promoting food over the years. The influence of long-term administration of *A. pullulans*-cultured fluid to the human health has been tested and reported by the Ministry of Health, Labour and Welfare in Japan. The report shows that any negative effects to the health have not been found by the long-term administration of *A. pullulans*-cultured fluid. Therefore, the systemic effects of oral administration of AP-CF on immunity are thought to be mild, and might be difficult to detect as a drastic change. Conversely, the fact that oral administration of AP-CF exhibits efficacy against an influenza A virus infection without strong systemic effects on immunity, would indicate the safety and the excellence of the *A. pullulans*-cultured fluid as a health-promoting food.

Our results using cultured cells show that the activity of AP-BG for immune stimulation is transient (Figure 4 and 5). However, β-(1→3),(1→6)-D-glucans are known to be a dietary fiber, and to remain in the small intestine for a long time without absorption. It has been reported this type of glucan, orally administered activates immunocompetence through the modulating Peyer's patch cells [47]. Therefore, it is assumed that the orally administered AP-CF constitutively activate the immunity by daily feedings. Our previous study using cattle suggested that the beneficial effects of the *A. pullulans*-cultured fluid on the health including the activation of immune system would appear after the long-term administration under conventional condition [48]. Although the properties of the effects of long-term administration of AP-CF on the immune system might be distinct from that of the short-term administration, the immunostimulating effects of AP-CF are not thought to be disappeared after the long-term administration.

Type I interferons (IFNs), such as IFNα and IFNβ, are responsible for activating the host-cellular anti-viral response. There are two major pathways for the production of type I IFNs through recognition of pathogens, one is the TLR (toll-like receptor) pathway which recognizes extracellular pathogens at the cell surface or endosome, and the other is the RLR (RIG-I-like receptors) pathway for recognition of intracellular pathogens [49], [50]. TLR7 and RIG-I (retinoic acid-inducible gene I) are known to be pattern recognition receptors which are responsible for recognition of influenza viruses [51], [52]. These receptors are crucial to activate the immune system for virus elimination through production of type I IFNs and inflammatory cytokines. TLR7 recognizes the viral single-stranded RNAs at endosome, and the activation of the TLR7-mediated signaling pathway for virus elimination depends on the function of MyD88, the downstream adaptor molecule. On the other hand, RIG-I recognizes the viral single-stranded RNAs bearing 5′-triphosphate in cytoplasm, and requires IPS-1, a mitochondrial adaptor molecule, for the activation of downstream signaling pathways. The responsibility of these intracellular or extracellular receptors to the production of type I IFN depends on the type of cells. In plasmacytoid dendritic cells (pDCs), The TLR7-mediated signaling pathway is predominantly responsible to type I IFN production [53], [54], and the RIG-I mediated pathway is crucial for its production in conventional dendritic cells (cDCs), alveolar macrophages (AMs) and fibroblasts [55], [56]. The previous report demonstrated that the AMs are important for the initial response for type I IFN production in the mouse lungs infected with an RNA virus [57]. This may indicate the significance of our findings for in vitro experiments using macrophage-derived RAW264.7 cells and macrophages, differentiated from THP-1 cells. As shown in Figure 5, the results demonstrated that expression of RIG-I and MDA5 is transiently increased after the treatment with *A. pullulans*-produced purified β-(1→3),(1→6)-D-glucan (AP-BG) in RAW264.7 cells and PMA-treated THP-1 cells. These observations suggest that oral administration of AP-CF increases the sensitivity of AMs to the influenza A virus infection through induction of RIG-I expression.

Previous reports indicated that nonstructural protein 1 (NS1) and viral RNA polymerase of influenza A virus are involved in the inhibition of the RIG-I mediated induction pathway for type I IFNs [58], [59]. However, RAW267.4 cells treated with AP-BG significantly inhibited replication of the influenza A virus (Figure 5D). Comparable results have been reported by using lung epithelial cell-derived A549 cells [45]. The report demonstrated that the treatment of tumor necrosis factor α (TNF-α) enhances RIG-I expression and inhibits replication of the influenza A virus through production of the anti-viral cytokines in A549 cells. These findings indicate that initial response of the production of type I IFNs after the virus infection is crucial for virus elimination. Since, no significant difference in the production of inflammatory cytokines and type I IFN (data not shown) was detected in our experimental conditions (Figure 2), the specific local activation of innate immunity without significant effects to the systemic immunity by the oral administration of AP-CF might be crucial for protection from a lethal influenza A virus infection.

Thus far, we focused on the specific protective effects of the oral administration of AP-CF against the influenza A virus infection on a possible mechanism for the regulation of the function of macrophages in vitro. In our in vivo mouse model, no apparent effect on immunity by oral administered AP-CF has been detected. Therefore, more detailed analysis in local immune response may be required for understanding the mechanism to exhibit the protective effects against the influenza A virus infection by oral administration of AP-CF. Since, interaction between AP-CF and influenza A virus particles has not been detected (data not shown), the mechanism for the efficacy of orally administered AP-CF against the influenza A virus infection is assumed to be of an indirect manner. Therefore, the oral administration of AP-CF might have a potential to exhibit efficacy for other infectious diseases caused by RNA viruses. Further investigations are required to understand the mechanism of the immune modulating function of the *A. pullulans*-produced β-(1→3),(1→6)-D-glucan.

## Materials and Methods

### Cell culture

A murine macrophage-like cell line, RAW264.7 (ATCC TIB-71) [25], and a human monocyte derived cell line, THP-1 (ATCC TIB-202) [26], [27] were cultured in RPMI-1640 medium (Sigma, St. Louis, MO) supplemented with 10% fetal bovine serum, 100 U/ml penicillin, and 100 mg/ml streptomycin, and was incubated at 37°C in 5% CO_2_. For differentiation of THP-1 into macrophages, THP-1 cells were treated with 100 nM of phorbol 12-myristate 13-acetate (PMA, Sigma) for 3 days. The cells were incubated without PMA for additional 24 hrs, and then used for the study.

### Preparation of Aureobasidium pullulans cultured fluid (AP-CF) and purified A. pullulans-produced β-(1→3),(1→6)-D-glucan (AP-BG)

*A. pullulans* was grown at 24.5°C for 10 days, in a medium containing rice bran and sucrose, as a nitrogen and a carbohydrate source respectively. After the cultured medium was heated at 90°C for 30 min, the heat-sterilized cultured medium was then diluted with PBS to the concentration of 2 mg/ml of β-(1→3),(1→6)-D-glucan, and used as AP-CF in this study.

For preparation of purified *A. pullulans*-produced β-(1→3),(1→6)-D-glucan (AP-BG), AP-CF was subjected to diatomaceous earth filtration to remove the cell debris, subsequently low molecular weight components were removed by adsorption using powdered activated carbon (Wako, Osaka, Japan), and by ultrafiltration with a cut-off molecular weight of 20,000 (Q2000; Advantec, Tokyo, Japan). The concentrated β-(1→3),(1→6)-D-glucans were then precipitated with ethanol at the concentration of 80%, and used for this study.

### Viral strain and titration

The A/Puerto Rico/8/34 (PR8; H1N1) strain of influenza A virus which was propagated in 10-day-old embryonated chicken eggs, was used for the experimental infection to mice. For the infection to cultured cells, the PR8 virus was propagated in MDCK cells at 35°C for 2 days, and the supernatant was used. Plaque assay for monitoring virus titers of lung homogenates was performed as described previously [60].

### Challenge the virus to mice

C57BL/6N mice (8-week-old, male) were purchased from Clea Japan Inc. (Tokyo, Japan), were orally administered with the β-glucan (2 mg/ml, 0.2 ml/mouse) or PBS through a syringe fitted with a ball-type feeding needle at once a day for 7 days, and subsequently were intranasally infected with the 2,000 pfu of PR8 strain of influenza A virus. After the infection, these mice were continued the administration with β-glucan at once a day, and clinical appearances were observed until the day 17.

All animal experiments were performed in accordance with the guidelines of the Bioscience Committee of Hokkaido University and were approved by the Animal Care and Use Committee of Hokkaido University.

### Flow cytometry

The antibodies, FITC-conjugated B220, Gr1 and CD49, APC-conjugated CD3e and CD11c, and PE-Cy7-conjugated F4/80 and CD69 were purchased as commercially available products (BD Bioscience, San Jose, CA).

The whole lung tissues were extracted from the mice infected with PR8 strain of influenza A virus at the day indicated in the figure. The lungs were minced, and incubated in Hank's balanced salt solution (HBSS; Invitrogen, Carlsbad, CA) containing 1 mg/ml collagenase D (Roche Diagnostics, Mannheim, Germany) and 0.02 mg/ml DNase I (Roche Diagnostics) at 37°C for 60 min. The dissociated lung cells were passed through a 70 µm nylon cell strainer (BD Bioscience), and then stained with an appropriate combination of antibodies. After staining for the dead cells with 7-AAD (BD Biosciences), the cells were analyzed by FACS (FACS Canto; BD bioscience).

### Real-time PCR analysis

The total RNA extractions from cultured cells and frozen mouse tissues were performed using TRIzol reagent (Invitrogen, Carlsbad, CA) according to the manufacturer's protocol. After the purity of the extracted RNAs was checked by agarose gel electrophoresis, the isolated RNAs were treated with DNaseI (Takara, Otsu, Japan), and then cDNAs were synthesized from the total RNAs by reverse transcription using ReverTraAce (Toyobo, Osaka, Japan) priming with random hexamer and oligo (dT).

Real-time PCR for monitoring the mRNA expression of inflammatory cytokines was carried out using SYBR Premix Ex Taq II (Takara, Otsu, Japan), and measured by Mx3000P quantitative PCR System (Stratagene, La Jolla CA). The specific primer set for interleukin-1β (IL-1β), IL-6, tumor necrosis factor-α (TNF-α), GM-CSF, G-CSF and interferon-γ (IFN-γ) were purchased as commercially available products (Takara). The following specific primer sets for RIG-I, MDA5 and IPS-1 were designed and used in this study; RIG-I (sense primer: 5′- GCAGACATGGGATGAAATGA -3′, anti-sense primer: 5′- TCTTGCACTTTCCACACAGC -3′); MDA5 (sense primer: 5′- AGTGTCAGCTGCTTCGATGA -3′, anti-sense primer: 5′- ATTTGGTAAGGCCTGAGCTG -3′); IPS-1 (sense primer: 5′- AGGTCACAACATCCCTGACC -3′, anti-sense primer: 5′- GGTCTGGAGGAGTTGCTCTG -3′).

## References

[1] Akramiene D, Kondrotas A, Didziapetriene J, Kevelaitis E (2007). Effects of beta-glucans on the immune system.. Medicina (Kaunas).

[2] Chan GC, Chan WK, Sze DM (2009). The effects of beta-glucan on human immune and cancer cells.. J Hematol Oncol.

[3] Novak M, Vetvicka V (2009). Glucans as biological response modifiers.. Endocr Metab Immune Disord Drug Targets.

[4] Chaung HC, Huang TC, Yu JH, Wu ML, Chung WB (2009). Immunomodulatory effects of beta-glucans on porcine alveolar macrophages and bone marrow haematopoietic cell-derived dendritic cells.. Vet Immunol Immunopathol.

[5] Liu J, Gunn L, Hansen R, Yan J (2009). Yeast-derived beta-glucan in combination with anti-tumor monoclonal antibody therapy in cancer.. Recent Pat Anticancer Drug Discov.

[6] Shimizu K, Watanabe S, Watanabe S, Matsuda K, Suga T (2009). Efficacy of oral administered superfine dispersed lentinan for advanced pancreatic cancer.. Hepatogastroenterology.

[7] Zhou LD, Zhang QH, Zhang Y, Liu J, Cao YM (2009). The shiitake mushroom-derived immuno-stimulant lentinan protects against murine malaria blood-stage infection by evoking adaptive immune-responses.. Int Immunopharmacol.

[8] Queiroz LS, Nascimento MS, Cruz AK, Castro AJ, Moura Mde F (2010). Glucans from the Caripia montagnei mushroom present anti-inflammatory activity.. Int Immunopharmacol.

[9] Sugiyama A, Hata S, Suzuki K, Yoshida E, Nakano R (2010). Oral administration of paramylon, a beta-1,3-D-glucan isolated from Euglena gracilis Z inhibits development of atopic dermatitis-like skin lesions in NC/Nga mice.. J Vet Med Sci.

[10] Oh-hashi F, Kataoka T, Tsukagoshi S (1978). Effect of combined use of anticancer drugs with a polysaccharide preparation, Krestin, on mouse leukemia P388.. Gann.

[11] Mashiba H, Matsunaga K, Gojobori M (1979). Effect of immunochemotherapy with OK-432 and yeast cell wall on the activities of peritoneal macrophages of mice.. Gann.

[12] Chihara G, Hamuro J, Maeda Y, Arai Y, Fukuoka F (1970). Fractionation and purification of the polysaccharides with marked antitumor activity, especially lentinan, from Lentinus edodes (Berk.) Sing. (an edible mushroom).. Cancer Res.

[13] Yamamoto T, Yamashita T, Tsubura E (1981). Inhibition of pulmonary metastasis of Lewis lung carcinoma by a glucan, Schizophyllan.. Invasion Metastasis.

[14] Brown RG, Lindberg B (1967). Polysaccharides from cell walls of Aureobasidium (Pullularia) pullulans. I. Glucans.. Acta Chem Scand.

[15] Brown RG, Lindberg B (1967). Polysaccharides from cell walls of Aureobasidium (Pullularia) pullulans. II. Heteropolysaccharide.. Acta Chem Scand.

[16] Hamada N, Deguchi K, Ohmoto T, Sakai K, Ohe T (2000). Ascorbic acid stimulation of production of a highly branched ,beta-1,3-glucan by Aureobasidium pullulans K-1–oxalic acid, a metabolite of ascorbic acid as the stimulating substance.. Biosci Biotechnol Biochem.

[17] Kataoka-Shirasugi N, Ikuta J, Kuroshima A, Misaki A (1994). Antitumor activities and immunochemical properties of the cell-wall polysaccharides from Aureobasidium pullulans.. Biosci Biotechnol Biochem.

[18] Kimura Y, Sumiyoshi M, Suzuki T, Sakanaka M (2006). Antitumor and antimetastatic activity of a novel water-soluble low molecular weight beta-1, 3-D-glucan (branch beta-1,6) isolated from Aureobasidium pullulans 1A1 strain black yeast.. Anticancer Res.

[19] Yatawara L, Wickramasinghe S, Nagataki M, Takamoto M, Nomura H (2009). Aureobasidium-derived soluble branched (1,3–1,6) beta-glucan (Sophy beta-glucan) enhances natural killer activity in Leishmania amazonensis-infected mice.. Korean J Parasitol.

[20] Kimura Y, Sumiyoshi M, Suzuki T, Suzuki T, Sakanaka M (2007). Inhibitory effects of water-soluble low-molecular-weight beta-(1,3–1,6) d-glucan purified from Aureobasidium pullulans GM-NH-1A1 strain on food allergic reactions in mice.. Int Immunopharmacol.

[21] Taubenberger JK, Morens DM (2010). Influenza: the once and future pandemic.. Public Health Rep.

[22] McCaughey C (2010). Influenza: a virus of our times.. Ulster Med J.

[23] Boyd M, Clezy K, Lindley R, Pearce R (2006). Pandemic influenza: clinical issues.. Med J Aust.

[24] Lynch JP, Walsh EE (2007). Influenza: evolving strategies in treatment and prevention.. Semin Respir Crit Care Med.

[25] Raschke WC, Baird S, Ralph P, Nakoinz I (1978). Functional macrophage cell lines transformed by Abelson leukemia virus.. Cell.

[26] Tsuchiya S, Kobayashi Y, Goto Y, Okumura H, Nakae S (1982). Induction of maturation in cultured human monocytic leukemia cells by a phorbol diester.. Cancer Res.

[27] Tsuchiya S, Yamabe M, Yamaguchi Y, Kobayashi Y, Konno T (1980). Establishment and characterization of a human acute monocytic leukemia cell line (THP-1).. Int J Cancer.

[28] Van Reeth K, Nauwynck H, Pensaert M (1998). Bronchoalveolar interferon-alpha, tumor necrosis factor-alpha, interleukin-1, and inflammation during acute influenza in pigs: a possible model for humans?. J Infect Dis.

[29] Julkunen I, Sareneva T, Pirhonen J, Ronni T, Melen K (2001). Molecular pathogenesis of influenza A virus infection and virus-induced regulation of cytokine gene expression.. Cytokine Growth Factor Rev.

[30] Kaiser L, Fritz RS, Straus SE, Gubareva L, Hayden FG (2001). Symptom pathogenesis during acute influenza: interleukin-6 and other cytokine responses.. J Med Virol.

[31] Mok KP, Wong CH, Cheung CY, Chan MC, Lee SM (2009). Viral genetic determinants of H5N1 influenza viruses that contribute to cytokine dysregulation.. J Infect Dis.

[32] Kimura Y, Sumiyoshi M, Suzuki T, Suzuki T, Sakanaka M (2007). Effects of water-soluble low-molecular-weight beta-1, 3-D-glucan (branch beta-1, 6) isolated from Aureobasidium pullulans 1A1 strain black yeast on restraint stress in mice.. J Pharm Pharmacol.

[33] Yoon HS, Kim JW, Cho HR, Moon SB, Shin HD (2010). Immunomodulatory effects of Aureobasidium pullulans SM-2001 exopolymers on the cyclophosphamide-treated mice.. J Microbiol Biotechnol.

[34] Sakurai T, Hashimoto K, Suzuki I, Ohno N, Oikawa S (1992). Enhancement of murine alveolar macrophage functions by orally administered beta-glucan.. Int J Immunopharmacol.

[35] Ikewaki N, Fujii N, Onaka T, Ikewaki S, Inoko H (2007). Immunological actions of Sophy beta-glucan (beta-1,3–1,6 glucan), currently available commercially as a health food supplement.. Microbiol Immunol.

[36] Tumpey TM, Garcia-Sastre A, Taubenberger JK, Palese P, Swayne DE (2005). Pathogenicity of influenza viruses with genes from the 1918 pandemic virus: functional roles of alveolar macrophages and neutrophils in limiting virus replication and mortality in mice.. J Virol.

[37] Coleman JR (2007). The PB1-F2 protein of Influenza A virus: increasing pathogenicity by disrupting alveolar macrophages.. Virol J.

[38] Kim HM, Lee YW, Lee KJ, Kim HS, Cho SW (2008). Alveolar macrophages are indispensable for controlling influenza viruses in lungs of pigs.. J Virol.

[39] Panopoulos AD, Watowich SS (2008). Granulocyte colony-stimulating factor: molecular mechanisms of action during steady state and ‘emergency’ hematopoiesis.. Cytokine.

[40] Conti L, Gessani S (2008). GM-CSF in the generation of dendritic cells from human blood monocyte precursors: recent advances.. Immunobiology.

[41] Hercus TR, Thomas D, Guthridge MA, Ekert PG, King-Scott J (2009). The granulocyte-macrophage colony-stimulating factor receptor: linking its structure to cell signaling and its role in disease.. Blood.

[42] Ehninger A, Trumpp A (2011). The bone marrow stem cell niche grows up: mesenchymal stem cells and macrophages move in.. J Exp Med.

[43] Takeuchi O, Akira S (2008). MDA5/RIG-I and virus recognition.. Curr Opin Immunol.

[44] Yoneyama M, Onomoto K, Fujita T (2008). Cytoplasmic recognition of RNA.. Adv Drug Deliv Rev.

[45] Matikainen S, Siren J, Tissari J, Veckman V, Pirhonen J (2006). Tumor necrosis factor alpha enhances influenza A virus-induced expression of antiviral cytokines by activating RIG-I gene expression.. J Virol.

[46] Husser L, Alves MP, Ruggli N, Summerfield A (2011). Identification of the role of RIG-I, MDA-5 and TLR3 in sensing RNA viruses in porcine epithelial cells using lentivirus-driven RNA interference.. Virus Res.

[47] Hashimoto K, Suzuki I, Yadomae T (1991). Oral administration of SSG, a beta-glucan obtained from Sclerotinia sclerotiorum, affects the function of Peyer's patch cells.. Int J Immunopharmacol.

[48] Uchiyama H, Iwai A, Asada Y, Muramatsu D, Aoki S (2012). A small scale study on the effects of oral administration of the beta-glucan produced by aureobasidium pullulans on milk quality and cytokine expressions of Holstein cows, and on bacterial flora in the intestines of Japanese black calves.. BMC Res Notes.

[49] Kawai T, Akira S (2007). Antiviral signaling through pattern recognition receptors.. J Biochem.

[50] Kawai T, Akira S (2008). Toll-like receptor and RIG-I-like receptor signaling.. Ann N Y Acad Sci.

[51] Koyama S, Ishii KJ, Kumar H, Tanimoto T, Coban C (2007). Differential role of TLR- and RLR-signaling in the immune responses to influenza A virus infection and vaccination.. J Immunol.

[52] Wang JP, Bowen GN, Padden C, Cerny A, Finberg RW (2008). Toll-like receptor-mediated activation of neutrophils by influenza A virus.. Blood.

[53] Diebold SS, Kaisho T, Hemmi H, Akira S, Reis e Sousa C (2004). Innate antiviral responses by means of TLR7-mediated recognition of single-stranded RNA.. Science.

[54] Lund JM, Alexopoulou L, Sato A, Karow M, Adams NC (2004). Recognition of single-stranded RNA viruses by Toll-like receptor 7.. Proc Natl Acad Sci U S A.

[55] Yoneyama M, Kikuchi M, Natsukawa T, Shinobu N, Imaizumi T (2004). The RNA helicase RIG-I has an essential function in double-stranded RNA-induced innate antiviral responses.. Nat Immunol.

[56] Kato H, Sato S, Yoneyama M, Yamamoto M, Uematsu S (2005). Cell type-specific involvement of RIG-I in antiviral response.. Immunity.

[57] Kumagai Y, Takeuchi O, Kato H, Kumar H, Matsui K (2007). Alveolar macrophages are the primary interferon-alpha producer in pulmonary infection with RNA viruses.. Immunity.

[58] Mibayashi M, Martinez-Sobrido L, Loo YM, Cardenas WB, Gale M (2007). Inhibition of retinoic acid-inducible gene I-mediated induction of beta interferon by the NS1 protein of influenza A virus.. J Virol.

[59] Iwai A, Shiozaki T, Kawai T, Akira S, Kawaoka Y (2010). Influenza A virus polymerase inhibits type I interferon induction by binding to interferon beta promoter stimulator 1.. J Biol Chem.

[60] Takada A, Matsushita S, Ninomiya A, Kawaoka Y, Kida H (2003). Intranasal immunization with formalin-inactivated virus vaccine induces a broad spectrum of heterosubtypic immunity against influenza A virus infection in mice.. Vaccine.

